# Psychological Factors, Central Sensitization, and Insomnia as Potential Prognostic Factors for Short‐Term Pain and Disability in Patients With Frozen Shoulder: A Multicentric Prospective Single‐Cohort Study

**DOI:** 10.1155/prm/6298409

**Published:** 2026-04-13

**Authors:** Fabrizio Brindisino, Elena Silvestri, Alessio Fioretti, Fabrizio Pulina, Jacopo Conteduca, Santiago Navarro Ledesma, Germanna Medeiros Barbosa, Filip Struyf

**Affiliations:** ^1^ Department of Medicine and Health Science “Vincenzo Tiberio”, University of Molise, Campobasso, Italy, unimol.it; ^2^ Department of Orthopaedic Surgery, Concordia Hospital, Rome, Italy; ^3^ Department of Physiotherapy, Pulina Private Practice, Genova, Italy; ^4^ Department of Orthopaedic and Traumatology, Vito Fazzi Hospital, Lecce, Italy; ^5^ Department of Clinical Medicine and Public Health PhD Program, Faculty of Health Sciences, University of Granada, Granada, Spain, ugr.es; ^6^ Department of Physical Therapy, Faculty of Health Sciences, Campus of Melilla, University of Granada, Melilla, Spain, ugr.es; ^7^ School of Health Sciences of Trairi, Post-Graduate Program in Physical Therapy, Universidade Federal Do Rio Grande Do Norte, Natal, Rio Grande Do Norte, Brazil, ufrn.br; ^8^ Research Group Movant, Department of Rehabilitation Sciences and Physiotherapy, Faculty of Medicine and Health Sciences, University of Antwerp, Antwerp, Belgium, uantwerpen.be

**Keywords:** anxiety, avoidance, behavior, biopsychosocial, catastrophization, sleep-wake disorders

## Abstract

**Background:**

Identifying modifiable prognostic factors is essential for personalizing conservative interventions. Several psychological and systemic variables remain underexplored in the frozen shoulder (FS) population, particularly regarding their prognostic relevance for short‐term changes in pain and disability.

**Aim:**

To estimate the prognostic value of psychological factors, central sensitization, and sleep disturbances in predicting clinical outcomes after 3 months of standardized conservative treatment.

**Method:**

This multicenter, prospective cohort study was reported following the REMARK statement. Individuals were recruited between July and September 2025. Baseline demographic and clinical variables such as pain catastrophizing, state and trait anxiety, fear‐avoidance beliefs (for Work and Physical Activity), central sensitization signs and symptoms, and insomnia were collected at baseline as potential prognostic factors. Shoulder pain and disability (measured with the SPADI subscores) served as outcome variables. Patients underwent a standardized multimodal conservative management for 3 months. Univariable and multivariable linear regression analyses were performed to examine associations between potential prognostic factors and SPADI pain and disability scores at 3‐month follow‐up.

**Results:**

Eighty participants were included. Trait anxiety emerged as the sole significant prognostic factor for shoulder pain (exp (β) = 1.21, 95% CI 1.06–1.38), while state anxiety significantly predicted disability (exp (β) = 0.83, 95% CI 0.73–0.95). The removal of outliers during sensitivity analysis eliminated the statistical significance of all potential prognostic factors when SPADI pain was the outcome. Regarding disability, however, state anxiety remained a significant prognostic factor (exp(β) = 0.86; 95% CI: 0.74–0.99).

**Discussion and Conclusion:**

Baseline trait and state anxiety showed prognostic relevance for short‐term pain and disability outcomes in patients with FS receiving conservative care. Pain catastrophizing, fear‐avoidance behavior, central sensitization, and insomnia did not show significant associations in this cohort. These findings underscore the importance of incorporating psychological profiling, particularly anxiety assessment, into the clinical evaluation and prognostic reasoning for patients with FS.

## 1. Background

Frozen shoulder (FS) is a prevalent cause of shoulder pain and stiffness, primarily affecting middle‐aged individuals, with a typically insidious onset and symptoms that may persist for months or even years [1]. Clinically, FS is defined by progressive pain and a marked deficit in both active and passive glenohumeral range of motion in the absence of other identifiable shoulder pathologies [2]. The estimated prevalence of FS in the general population ranges from 2% to 5%, with a higher rate among women, sedentary individuals, and those aged 40–65 years [3].

Classically, the clinical course is described as three overlapping phases (i.e., freezing, frozen, and thawing). However, contemporary evidence challenges this linear model, as several studies have reported the absence of clearly defined phases or proposed alternative staging frameworks comprising two, three, or even four stages, underscoring the complexity of its clinical presentation [4]. This lack of consensus poses challenges for prognostic estimation and clinical decision‐making [4]. Although FS is often characterized by improvement, a substantial subset of individuals experiences lasting movement restrictions and residual pain [5]. For example, up to 40% of individuals report persistent symptoms or motion restriction for as long as 7 years post onset [5, 6].

In an effort to identify patients at risk of poor prognosis, recent literature has increasingly focused on the identification of FS’s prognostic factors, defined as patients’ characteristics that help predict the likely course or outcome of a disease. Identifying modifiable prognostic factors is essential to support personalized conservative interventions within a biopsychosocial management. As an example, patient‐related factors, such as diabetes mellitus, thyroid disorders, and bilateral presentation, are strong prognostic factors of poorer outcomes and may identify patients who need early surgical intervention [7–9]. Moreover, higher baseline pain and disability, severe motion restriction, and longer symptom duration have been linked to worse functional recovery [10, 11]. In parallel, psychological factors such as kinesiophobia, catastrophizing, anxiety, and poor health‐related quality‐of‐life scores have emerged as significant predictors of persistent pain and disability [10, 12, 13]. Nonetheless, anxiety was often considered as a unitary construct, without differentiating between trait and state anxiety associated with the concurrent condition, and some variables, including some psychosocial factors, central sensitization, and sleep disturbances, remain underexplored as prognostic indicators in patients with FS. It is worth noting that research in this field has historically emphasized biological and mechanical aspects, often overlooking broader dimensions of the patient experience [14, 15]. Although recent qualitative studies have begun to characterize the personal burden of FS, analyzing its impact on individual, occupational, and social environments [16–18], quantitative prognostic research addressing these domains remains limited and methodologically heterogeneous. Common limitations include the absence of a priori sample size calculations, suboptimal study designs (e.g., retrospective analyses), and insufficient control for confounding variables, contributing to inconsistent or preliminary findings [10, 11].

Therefore, this study aims to evaluate, with methodological rigor, the prognostic value of pain catastrophizing, state and trait anxiety, fear‐avoidance beliefs related to physical activity and work, central sensitization, and sleep disturbances in patients with FS, addressing the conflicting/unclear results and knowledge gaps found in recent literature [11]. Improving the understanding of these potentially modifiable factors may support future prognostic modeling efforts, inform personalized management, and facilitate more effective and empathetic communication regarding recovery expectations.

## 2. Methods

### 2.1. Study Design and Ethical Approval

This 3‐month prospective, multicenter, single‐cohort study was reported in accordance with the REporting recommendations for tumor MARKer prognostic studies (REMARK) statement [19]. Ethical approval for the study’s methods and procedures was obtained from the University of Molise (Italy) (Institutional Review Board registration number: 06/2025 from June 19, 2025). All study‐related procedures were conducted in compliance with the principles of the Declaration of Helsinki [20].

### 2.2. Setting, Recruitment of Individuals, and Inclusion and Exclusion Criteria

Individuals were recruited from three private physiotherapy practices located in Rome, Lecce, and Genoa (Italy) between July 2025 and September 2025.

The eligibility of individuals was assessed by four physiotherapists (ES, AF, FB, FP), each possessing over a decade of specialized experience in managing patients with shoulder musculoskeletal disorders. All screening physiotherapists adhered scrupulously to the inclusion and exclusion criteria reported below, and all individuals were screened during their initial direct‐access consultation for shoulder pain.

To be included in the study, individuals had to present with unilateral shoulder pain of nontraumatic origin. A primary clinical requirement was a significant deficit in shoulder mobility, specifically a passive and active external rotation, with the arm at the side, restricted to less than 50% of the contralateral side, as well as a range of motion restriction of at least 25% was required in two additional planes of movement [2]. To reduce the likelihood of spontaneous early recovery, symptoms were required to be stable or worsening over the preceding month [2]. Finally, a negative radiographic evaluation for significant glenohumeral osteoarthritis was mandatory; only Grade 1 on the Kellgren–Lawrence scale [21] was permitted to ensure the sample accurately represented individuals with FS rather than joint degeneration [22]. A musculoskeletal radiologist at each private practice, who was blinded to the study objectives, classified the grade of osteoarthritis for all included participants.

Exclusion criteria were applied to minimize confounding. These included prior medical consultations for the same episode of shoulder pain or symptoms suggestive of cervical involvement (e.g., pain reproduced by neck movements) [23], or positive findings on the Wainner clinical cluster for cervical radiculopathy [24]. Individuals with a history of major trauma or surgical intervention on the affected shoulder within the last 5 years were also excluded. Imaging‐based exclusion criteria included the presence of significant rotator cuff calcific tendinopathy (calcifications > 2 mm) [21].

Eligible individuals received detailed information regarding the research aims and procedures via an information letter (Supporting file 1), and written informed consent was obtained prior to participation. Individuals who declined to participate were directed to standard rehabilitative care for their condition.

### 2.3. Variables and Measurements (Assay Methods)

#### 2.3.1. Demographics and Clinical History

Data were collected on age, gender, arm dominance, affected side, history of previous FS, occupation, educational level, and physical activity (including whether such activity involved the affected shoulder). Comorbidities screening included diabetes, hyperglycemia, thyroid disorders, Dupuytren’s contracture, cardiopulmonary, rheumatic, autoimmune, psychiatric/psychological, and neurological diseases, and neoplasms. Current pharmacotherapy intake was also monitored. Clinical information regarding daytime, nocturnal, and activity‐related pain and stiffness was also recorded.

#### 2.3.2. Outcomes (Dependent Variables)

Given that pain and disability represent the primary concerns for individuals affected by FS [25, 26], the Shoulder Pain and Disability Index (SPADI) was selected as the primary dependent variable for this study.

The SPADI comprises 13 items categorized into two distinct domains: a 5‐item pain subscale and an 8‐item disability subscale [27]. These subscales specifically measure the challenges individuals face during routine activities, such as lifting objects, reaching, or sleeping. Participants rate each item on a scale from 0 to 10, with higher scores reflecting increased symptom severity or functional impairment. For the current investigation, the Italian version of the SPADI was employed [28], as it has demonstrated feasibility, reliability, and validity in patients with shoulder disorders. Specifically, the cross‐culturally adapted and validated Italian version of the SPADI has demonstrated satisfactory construct validity, sound results in confirmatory factor analysis, excellent internal consistency, and high test–retest reliability for both pain and disability subscores [29].

Participants independently completed the SPADI, provided in a dedicated booklet, without any clinical assistance to ensure unbiased responses. To maintain participant anonymity while allowing for data integration, each booklet was assigned a unique chronological code. This system enabled the matching of patient‐reported outcomes with demographic and clinical history data and with the independent variables scores without revealing the identity of the individuals. The SPADI was administered via Google Forms (https://docs.google.com/forms) at two specific time points: the initial consultation (baseline) and at the 3‐month follow‐up, which represented the predefined end of the observation period. Indeed, although “recovery” was not operationally defined, a 12‐week follow‐up period was selected based on evidence, indicating that the clinical course of FS typically exhibits rapid improvement during the first three months, followed by a deceleration in progress thereafter [30, 31].

#### 2.3.3. Candidate Prognostic Factors (Independent Variables)

The independent variables investigated for the study aim were as follows:

• Pain catastrophizing measured with the *Pain Catastrophizing Scale (PCS).* The PCS is a 13‐item scale to quantify negative thoughts that may be experienced in the presence of pain. Each question is scored on a 5‐point Likert scale (from “not at all” to “always”). The total score of the PCS ranges between 0 and 52 with a higher score indicating greater pain catastrophizing. The Italian version of the PCS was used in the present study [32].
• State and trait anxiety measured with the *State–Trait Anxiety Inventory (STAI-T and STAI-S).* The STAI is a 40‐item multiple‐choice self‐report inventory divided into two subtests, each having 20 items (for both state and trait anxiety; ‐S and ‐T, respectively), aimed at assessing and quantifying anxiety disorders in adults. All items are rated on a 4‐point Likert scale, and higher scores reflect a higher level of disorder. The Italian version of the STAI was adopted for this study [33].
• Fear‐avoidance beliefs measured with the *Fear-Avoidance Beliefs Questionnaire (FABQ).* The FABQ has a 2‐factor, 12‐item structure and is reliable, valid, and responsive. It can therefore be recommended for clinical and research purposes because it is expected to improve the cognitive‐behavioral assessment. The 2 factors were called FABQ‐work and FABQ‐physical activity. The subscores are 0–42 for FABQ‐work, and 0–30 for FABQ‐physical activity, with a higher value reflecting a higher degree of fear‐avoidance beliefs. The culturally adapted Italian version of the FABQ was used in this study [34].
• Central sensitization measured with the *central sensitization inventory (CSI).* The CSI is a patient‐reported instrument designed to identify individuals’ signs and symptoms related to central sensitization. It consists of two parts: Part A provides a 0–100 total score for 25 items on current health symptoms with five response options ranging from “never” (0) to “always” [4]; Part B investigates if patients were previously diagnosed by a physician with one or more of seven different conditions. The Italian cross‐culturally adapted version of the CSI was used in this study [35].
• Insomnia measured with the *Insomnia Severity Index (ISI).* ISI is a seven‐item questionnaire that assesses, during the previous 2 weeks: the severity of sleep onset (Item 1a), the severity of sleep maintenance (Item 1b), early morning awakenings (Item 1c), satisfaction level with current sleep pattern (Item 2), interference with daily living (Item 3), noticeability of impairment due to the sleep difficulty (Item 4), and level of distress caused by the sleep problem (Item 5). Each item is rated on a 5‐point Likert scale of 0–4 (for Items 1–3, 0 = no problem, 4 = very severe problem; for Item 4, 0 = very satisfied, 4 = very dissatisfied; for Items 5–7, 0 = not at all, 4 = very much), and the total score ranges from 0 to 28, with a higher score indicating greater insomnia severity. The Italian version of the ISI was adopted for this study [36].

These questionnaires were further administered at baseline and in an anonymous manner without any clinical assistance. To maintain participant anonymity while allowing for data integration, each booklet was assigned a unique chronological code that matched other variables collected without revealing the identity of the individuals. A Google Form link was also used to administer these questionnaires.

### 2.4. Clinical Management During Cohort Time

Following completion of the baseline assessment, all participants received standard clinical care for FS during the follow‐up period. The clinical management described below was not considered an exposure of interest, but rather reflects usual physiotherapy care provided to all participants to contextualize outcome interpretation. All participants attended an educational session featuring standardized content across centers. This session addressed the natural history of FS, predisposing and prognostic factors, management options, home exercise recommendations, and expected recovery timelines.

After the initial consultation, participants were referred to a qualified clinician for pharmacological management. In accordance with clinical expertise and personal preference, the clinician determined whether to provide oral corticosteroids or landmark‐guided intra‐articular injections. All individuals received an initial pharmacological intervention; subsequent administrations were provided upon reasonable individual request (e.g., in cases of unbearable daytime or nocturnal pain). Additionally, participants were permitted to use nonsteroidal anti‐inflammatory drugs on an as‐needed basis.

Physical therapy rehabilitation commenced within five days of the initial consultation and consisted of joint mobilization [37], exercise therapy [38], and stretching [39], complemented by detailed instructions for a daily 30‐min home exercise program. Supervised sessions lasted approximately 45 min and were scheduled twice weekly during the first 2 weeks, once weekly for the subsequent 6 weeks, and once every 2 weeks during the final month. In all clinical settings, care was delivered by physiotherapists with over 12 years of experience in shoulder pathologies, who adjusted manual techniques and home prescriptions according to individual tissue irritability levels [40].

### 2.5. Individuals’ Completion/Withdrawal

Participants were free to withdraw from the study at any time without prejudice to their clinical care. They may also be discontinued from the study at the discretion of the investigator for lack of adherence to study treatment or visit schedules, adverse events (AEs), or any other reason (to be documented). In addition, physiotherapists involved in the clinical management of participants could withdraw individuals who violate the study plan, or when withdrawal was deemed necessary to ensure participant safety or for administrative reasons.

### 2.6. Clinical AEs

Clinical AEs were monitored throughout the study and assessed at 3 months. An AE is any untoward medical occurrence in an individual who has received an intervention (e.g., drug, biological, or other interventions) [41]. The occurrence does not necessarily have to have a causal relationship with the treatment. An AE can therefore be any unfavorable or unintended sign (including an abnormal laboratory finding, for example), symptom, or disease temporally associated with using a medicinal product, whether considered related to the medicinal product. All AEs (including serious AEs) were noted in the study records and on the case report form with a complete description including the nature, date and time of onset, determination of nonserious versus serious, intensity (mild, moderate, and severe), duration, causality, and outcome of the event. If the physiotherapists became aware of any serious, related AEs after the individual completes or withdraws from the study, such events were recorded.

### 2.7. Sample Size Calculation

As there are no closed‐form formulas for sample size calculation in studies evaluating prognostic factors [42], the sample size rationale was based on a commonly used rule of thumb recommending at least 10 observations per regression coefficient [43]. Given that the multivariable models included eight regression coefficients, a sample of 80 participants was considered sufficient to fit them.

### 2.8. Statistical Analysis

Descriptive statistics were calculated to summarize participants’ baseline characteristics and outcome measures.

Univariable and multivariable linear regression analyses were conducted to examine the prognostic associations between potential prognostic factors and the outcomes of interest, namely, pain and disability at 3‐month follow‐up. The same set of baseline variables—PCS, STAI‐T, STAI‐S, FABQ‐physical activity, FABQ‐work, CSI, and ISI—was included in both univariable and multivariable analyses, with all potential prognostic factors entered simultaneously into the multivariable models. Moreover, baseline SPADI pain was included as a confounding variable in the model where SPADI pain was the outcome, while baseline SPADI disability was included in the model where SPADI disability was the outcome.

Model assumptions were assessed using standard diagnostic procedures. Linearity, homoscedasticity, and normality of residuals were evaluated through visual inspection of residuals‐versus‐fitted plots, scale–location plots, and normal Q–Q plots. Multicollinearity among potential prognostic factors was assessed using variance inflation factors, with values greater than 5 indicating potentially problematic collinearity. Finally, influential observations were identified using Cook’s distance, with values exceeding 4/*n* (where *n* is the sample size) considered potentially influential.

Had missingness occurred, it would have been handled using multiple imputation by chained equations.

Preliminary model diagnostics indicated violations of normality and homoscedasticity in the residuals of models fitted using the original outcome scales. Consequently, SPADI pain and SPADI disability scores were log‐transformed prior to regression analyses. Regression coefficients from the log‐transformed models were exponentiated and are presented as ratios with corresponding 95% confidence intervals. These exponentiated coefficients can be interpreted as the relative change (multiplicative effect) in the outcome associated with a one‐unit increase in the prognostic factor, holding all other variables constant.

A sensitivity analysis was performed by refitting the multivariable models after excluding observations identified as influential. All analyses were conducted using R (R Foundation for Statistical Computing, Vienna, Austria) with the readxl, Table 1, dplyr, officer, car, and lmtest packages.

**TABLE 1:** Demographic and clinical information of the recruited sample at baseline (*n* = 80).

**Variable**	

*Age*	
Mean (SD)	55.3 (9.6)
Median [Min, Max]	53.5 [34.0, 82.0]

*Sex*	
Female	57 (71.3%)
Male	23 (28.8%)

*Occupational Status*	
Self‐employed	7 (8.8%)
Homemaker	3 (3.8%)
Salaried employee	58 (72.5%)
Retired	12 (15.0%)

*Education (Years)*	
Mean (SD)	15.5 (4.4)
Median [Min, Max]	17.0 [5.0, 25.0]

*Physical Activity*	
No	39 (48.8%)
Yes	41 (51.3%)

*Shoulder-specific Physical Activity*	
No	47 (58.8%)
Yes	33 (41.3%)

*Dominant Arm*	
Right	76 (95.0%)
Left	4 (5.0%)

*Affected Arm*	
Right	45 (56.3%)
Left	35 (43.8%)

*Previous FS (Ipsilateral)*	
No	71 (88.8%)
Yes	9 (11.3%)

*Previous FS (Contralateral)*	
No	64 (80.0%)
Yes	16 (20.0%)

*Comorbidities*	
Diabetes mellitus	8 (10.0%)
Hyperglycemia	6 (7.5%)
Thyroid disorders	13 (16.3%)
Dupuytren’s contracture	0 (0%)
Cardiopulmonary diseases	0 (0%)
Rheumatic diseases	6 (7.5%)
Dysmetabolic disorders	5 (6.3%)
Autoimmune	11 (13.8%)
Neoplasms	6 (7.5%)
Neurological	2 (2.5%)
Shoulder tumors	0 (0%)
Shoulder infections	0 (0%)
Psychiatric	0 (0%)
Psychological	7 (8.8%)

*Pharmacotherapy*	
Anxiolytics/antidepressants	4 (5.0%)
Barbiturates	0 (0%)
Analgesics (painkillers)	17 (21.3%)
Muscle relaxants	3 (3.8%)
NSAIDs	16 (20.0%)

*Baseline Pain and Stiffness (0–10 NRS)*	
Diurnal pain (Mean, SD)	4.2 (2.6)
Nocturnal pain (Mean, SD)	4.8 (2.8)
Pain during activity (Mean, SD)	5.8 (2.3)
Stiffness (Mean, SD)	5.8 (2.6)

*Note:* Data are presented as mean (standard deviation), median [minimum, maximum], or number (percentage), as appropriate.

Abbreviations: FS, frozen shoulder; NRS, numeric rating scale; NSAIDs, nonsteroidal anti‐inflammatory drugs; SD, standard deviation.

## 3. Results

### 3.1. Demographics

**FIGURE 1:**
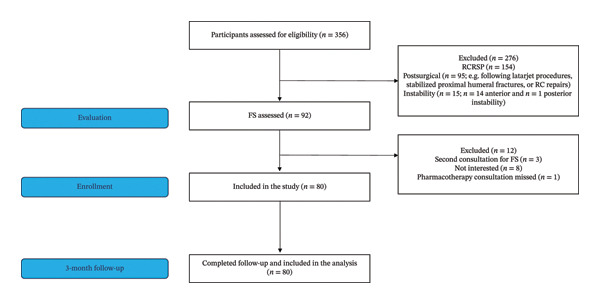
Flowchart of the study. Abbreviations: RCRSP, rotator cuff‐related shoulder pain; RC, rotator cuff; FS, frozen shoulder.

During the recruitment period, a total of 356 individuals presented for consultation across the three clinical settings due to shoulder disorders. Of these, 92 individuals presented with a primary complaint of FS. Among this group, three individuals were excluded as they were attending a second consultation, and eight expressed no interest in the investigation and declined to provide informed consent. One individual missed the consultation for pharmacotherapy administration and was further excluded. Consequently, a final sample of 80 participants was recruited for the study (Figure 1).

All included participants provided written informed consent and completed the full battery of assessments at both the initial consultation and the 3‐month follow‐up, resulting in a 100% completion rate. No missing data were observed for patient‐reported outcome measures for predictors and outcomes, and no participants missed scheduled rehabilitation appointments. The mean time required to complete the assessment booklet was 16.22 min. Clinically, all participants were classified by the treating physiotherapists as presenting moderate to high tissue irritability [40], and clinical management was adjusted accordingly.

No major AEs were reported during the study period. Five participants reported transient local pain at the injection site (3/10 numeric rating scale for pain) which resolved spontaneously within 24 h. Although all participants received an initial corticosteroid injection, only 12 required a second dose, and four required a third. No concomitant oral pharmacological therapy was administered, and the use of nonsteroidal anti‐inflammatory drugs was not investigated.

The recruited sample primarily consisted of right‐handed, female employees, with a mean age of 55.3 years (±9.57). While the majority of the sample engaged in physical activity, participants reporting sports that involve shoulder‐specific activity were less represented. The right arm was the most frequently affected side, and most participants reported this as their first episode of FS. Regarding comorbidities, thyroid disorders, autoimmune diseases, and diabetes mellitus were the most frequently reported conditions. Personal pharmacological management primarily involved the use of analgesics and nonsteroidal anti‐inflammatory drugs. Detailed demographic information and patient‐reported outcome measures values are summarized in Tables 1 and 2, respectively.

**TABLE 2:** Patient‐reported outcome measures collected (*n* = 80).

**Construct measured**	**Independent variables**	**Range (min, max)**	**Mean, SD**

Pain catastrophizing	PCS^a^	0–52^c^ ^,^ ^d^	26.0 (24.1)
Trait anxiety	STAI‐trait^a^	20–80^c^ ^,^ ^e^	44.4 (6.9)
State anxiety	STAI‐state^a^	20–80^c^ ^,^ ^f^	45.0 (9.2)
Fear‐avoidance beliefs	FABQ‐physical activity^a^	0–30^c^ ^,^ ^g^	12.2 (6.6)
Fear‐avoidance beliefs	FABQ‐work^a^	0–42^c^ ^,^ ^h^	10.4 (12.0)
Central sensitization	CSI^a^	0–100^c^ ^,^ ^i^	27.8 (14.3)
Insomnia	ISI^a^	0–28^c^ ^,^ ^l^	9.1 (6.5)

**Construct measured**	**Dependent variables**	**Range (min, max)**	**Mean, SD**

Shoulder pain	SPADI pain^a^	0–50^c^	56.5 (21.7)
Shoulder disability	SPADI disability^a^	0–80^c^	49.0 (21.9)
Shoulder pain	SPADI pain^b^	0–50^c^	23.7 (22.8)
Shoulder disability	SPADI disability^b^	0–80^c^	18.9 (22.4)

Abbreviations: CSI, central sensitization inventory; FABQ, Fear‐Avoidance Beliefs Questionnaire; ISI, Insomnia Severity Index; PCS, pain catastrophizing scale; SD, standard deviation; SPADI, Shoulder Pain and Disability Index; STAI, state–trait anxiety inventory.

^a^baseline administration.

^b^follow‐up administration.

^c^higher scores reflect increased severity.

^d^score > 30 means clinically significant catastrophizing.

^e^score > 40 means a stable personality trait of high anxiety.

^f^score > 40 means clinically significant current anxiety symptoms.

^g^score > 15 means high fear‐avoidance beliefs regarding movement.

^h^score > 34 means high risk for prolonged work disability.

^i^score > 40 means the presence of central sensitivity syndrome.

^l^score > 15 means clinically significant moderate‐to‐severe insomnia.

Both the SPADI pain and disability subscales demonstrated remarkable positive changes at follow‐up, with within‐group score reductions exceeding 50%. Although a specific minimal important change has not been established for the individual subscales in the FS literature, this substantial decrease strongly suggests that the conservative treatment achieved a profound and meaningful enhancement in both functional capacity and symptomatic relief.

### 3.2. Regression Analysis

In multivariable models for both outcomes, all VIF values were below the threshold of 5, indicating the absence of relevant multicollinearity among the potential prognostic factors (Supporting file 2). Detailed results are presented in Tables 3 and 4, for pain and disability, respectively.

**TABLE 3:** Regression analysis for potential prognostic factors and pain outcome at 3‐month follow‐up (*n* = 80).

Variable	Univariable analysis		Multivariable analysis	
	exp (coefficients)	*p* value	exp (coefficients)	*p* value
PCS	1.01 (0.99–1.03)	0.46	1.00 (0.97–1.02)	0.82
STAI‐trait anxiety	**1.09 (1.02 to 1.16)**	**0.01**	**1.21 (1.06 to 1.38)**	**< 0.01**
STAI‐state anxiety	1.03 (0.98–1.08)	0.18	0.92 (0.83–1.01)	0.08
FABQ‐physical activity	1.07 (1.00–1.14)	0.06	1.06 (0.97–1.17)	0.20
FABQ‐work	1.01 (0.98–1.05)	0.49	0.98 (0.93–1.03)	0.43
CSI	1.02 (0.99–1.06)	0.13	1.00 (0.95–1.04)	0.84
ISI	1.05 (0.98–1.12)	0.16	1.01 (0.91–1.12)	0.86

*Note:* Significant values in bold.

Abbreviations: CSI, central sensitization inventory; FABQ, Fear‐Avoidance Beliefs Questionnaire; ISI, Insomnia Severity Index; PCS, pain catastrophizing scale; STAI, state–trait anxiety inventory.

**TABLE 4:** Regression analysis for potential prognostic factors and disability outcome at 3‐month follow‐up (*n* = 80).

Variable	Univariable analysis		Multivariable analysis	
	exp (coefficients)	*p* value	exp (coefficients)	*p* value
PCS	1.0 (0.97–1.03)	0.75	0.99 (0.96–1.03)	0.64
STAI‐trait anxiety	0.98 (0.89–1.09)	0.75	1.17 (0.98–1.40)	0.08
STAI‐state anxiety	0.94 (0.87–1.02)	0.15	**0.83 (0.73 to 0.95)**	**< 0.01**
FABQ‐physical activity	1.03 (0.92–1.15)	0.56	1.05 (0.93–1.20)	0.41
FABQ‐work	1.01 (0.95–1.07)	0.83	0.97 (0.90–1.05)	0.45
CSI	1.03 (0.98–1.09)	0.20	1.00 (0.94–1.07)	0.98
ISI	1.05 (0.94–1.17)	0.38	1.03 (0.89–1.19)	0.72

*Note:* Significant values in bold.

Abbreviations: CSI, central sensitization inventory; FABQ, Fear‐Avoidance Beliefs Questionnaire; ISI, Insomnia Severity Index; PCS, pain catastrophizing scale; STAI, state–trait anxiety inventory.

In the multivariable model for SPADI pain, *trait* anxiety was the only variable that showed a statistically significant association with pain scores at follow‐up (Table 3). None of the other investigated variables demonstrated statistically significant associations with SPADI pain at 3‐month follow‐up.

Regarding SPADI disability, *state* anxiety was the only variable independently associated with disability scores at follow‐up in the multivariable analysis (Table 4). No statistically significant associations were observed for the other potential prognostic factors.

### 3.3. Outlier Analysis

Outlier analysis (Supporting file 3) revealed the presence of influential observations. Regarding the SPADI pain subscale, sensitivity analyses excluding outliers showed that none of the variables remained statistically significant in the multivariable models. Conversely, for SPADI disability, state anxiety remained a significant prognostic factor (exp (β) = 0.86; 95% CI: 0.74–0.99).

## 4. Discussion

This study examined the prognostic value of pain catastrophizing, state and trait anxiety, fear‐avoidance beliefs regarding physical activity and work, central sensitization, and insomnia on pain and disability outcomes in individuals with FS undergoing conservative and pharmacological management. Our findings revealed that trait anxiety was the sole significant prognostic factor for pain, while state anxiety was the only prognostic factor significantly associated with disability. Moreover, the clinical relevance of the observed changes in the individual SPADI subscales could not be fully established, as minimal important changes for the SPADI pain and disability subscores have not been reported in the FS literature.

### 4.1. Relation to Previous Studies

These findings are consistent with previous evidence highlighting the role of anxiety in both baseline clinical presentation and prognosis in individuals with FS. In fact, a higher anxiety score has been associated with worse scores in pain and disability [14, 44], confirming the importance of this psychological variable on the clinical score of primary interest. Furthermore, our findings support the conclusions of a recent systematic review, which identified a significant association between anxiety and both pain and disability at a 3‐month follow‐up [12], and another one that indicated anxiety as a variable co‐responsible for a change in functional score assessed with Patient‐Reported Outcomes Measurement Information System—Upper Extremity (PROMIS‐UE) [45]. Importantly, by utilizing the STAI, this multicenter prospective study confirms these associations within a robust methodological framework and extends the current literature by differentiating, for the first time in the field of FS research, the specific contributions of “trait” versus “state” anxiety to different outcomes in the clinical course of the condition. *State* anxiety is defined as a transient emotional condition; a “snapshot” of a person’s feelings at a specific moment, often reacting to a localized stressor and subsiding once the threat passes. Conversely, *trait* anxiety is a stable personality characteristic reflecting a baseline tendency to perceive environments as threatening; it remains relatively constant over time and dictates a person’s general response to life stressors [46].

A notable finding of this study is that *STAI-Trait anxiety* emerged as a significant prognostic factor for pain at the 3‐month follow‐up. This distinction is clinically vital, as it suggests that dispositional characteristics, rather than a transient emotional response to the condition, may be more closely related to pain outcomes over time. In the context of FS, individuals with high trait anxiety may be “pre‐sensitized” to pain. This chronic state of heightened emotional vigilance can interfere with the resolution of the inflammatory phase, as persistent psychological arousal is known to modulate the body’s inflammatory environment [47, 48].

The biological transduction of external social and environmental experiences into internal physiological signals is well‐documented [48]. Neural representations of adversity, particularly within brain systems processing social and physical unpleasantness (e.g., the anterior insula and dorsal anterior cingulate cortex), project to lower level areas that modulate inflammatory activity via the hypothalamic–pituitary–adrenal axis, the sympathetic nervous system, and the efferent vagus nerve [48]. This cascade results in the production of proinflammatory cytokines, which signal the brain to induce cognitive and behavioral alterations (such as low mood, anhedonia, and sleep disturbances) often described as “sickness behavior.” These states are frequently observed in FS individuals, reinforcing the need for early psychological screening to identify patients requiring comprehensive, multidisciplinary support.

Our regression analysis also identified *STAI-State anxiety* as a significant correlate of disability severity. This suggests that the psychological distress regarding functional loss is also a reactive phenomenon, a transient response to acute pain, functional limitations, the struggle to resume normal activities, and the clinical uncertainty associated with the pathology, likely reflecting the immediate “threat” perceived during the early phase [16–18]. This strongly suggests that the emotional burden is a direct consequence of the disease’s impact on both body and mind, fundamentally altering the patient’s “sense of self” [16]. As a result, individuals often describe themselves as living in a “no‐man’s land” [17], caught between their previous functional identity and the limitations imposed by the pathology. Consequently, therapeutic strategies emphasizing patient education, clear prognostic communication, and reassurance regarding the self‐limiting nature of the pathology, as well as stress‐reduction techniques, are essential. Specific interventions, such as slow deep breathing, aerobic exercise, and relaxation techniques like yoga, can mitigate this situational anxiety [49]. By lowering the perceived threat level, clinicians can improve functional outcomes and enhance patient engagement with conservative rehabilitation protocols.

Personality‐related differences in stress reactivity may translate into interindividual variability in neuroimmune–immunometabolic signaling proposed to contribute to FS onset and/or perpetuation [50]. In turn, this variability could shape how patients perceive and respond to symptoms (e.g., perceived threat, avoidance, and engagement with rehabilitation), influencing pain‐related behavior and potentially the persistence of inflammatory and tissue‐remodeling processes. Therefore, assessing state anxiety together with personality profiles may improve patient phenotyping, support more personalized management, and help anticipate the likely response to conservative care [51–53].

Our study is the first to investigate the prognostic value of fear‐avoidance beliefs, central sensitization, and insomnia in the FS field, and our findings demonstrated nonsignificant prognostic results regarding such variables, as well as pain catastrophizing, in relation to short‐term pain and disability outcomes. This contrasts with some existing literature where a fair positive correlation over 9 months was found between pain intensity and pain catastrophizing in a longitudinal multicenter prospective observational study [54]. Moreover, pain catastrophizing has shown baseline associations with perceived arm function and pain intensity [14, 55–57], as well as with expanded pain distribution areas [58]. Furthermore, PCS has previously shown significant differences between FS individuals and healthy controls [49]. However, this variable did not reach significance as a longitudinal predictor in our cohort.

One explanation for this discrepancy may be related to differences in care delivery. In the longitudinal study by Mertens et al. (2023) [54], no standardized care pathway was implemented, and participants accessed care on an as‐needed basis. Conversely, our sample underwent a structured 3‐month multimodal program involving education, pharmacotherapy, exercise, joint mobilization, and stretching. It is plausible that this more comprehensive and consistent care context attenuated the longitudinal association between this psychological variable and pain and disability outcomes. However, the magnitude of change in shoulder pain and disability observed in the present study is consistent with previous reports [59–61], and it aligns with current evidence‐based clinical guidelines [62].

The lack of association between clinical outcomes and pain catastrophizing, fear‐avoidance beliefs, central sensitization, and sleep disturbance may be partly explained by the specific baseline psychological profile of the recruited sample. Overall, participants exhibited relatively low levels of these constructs (e.g., below the established clinical thresholds for “high risk”) at study entry, potentially limiting their variability and reducing the ability to detect longitudinal associations with pain and disability outcomes. In contrast, anxiety‐related measures appeared more prominent in this cohort, which may help explain why anxiety emerged as the primary psychological factor associated with clinical outcomes. This restricted variability in nonanxiety domains suggests a possible “floor effect,” whereby these variables may still be clinically relevant in other FS populations with a different baseline psychological burden. Therefore, our cohort may not be fully representative of patients with extreme levels of catastrophizing, kinesiophobia, central sensitization, or severe sleep disturbances.

### 4.2. Outlier Analysis

The findings of outliers’ analysis with SPADI pain as an outcome raise the possibility that the main results were distorted by the influence of outlying cases, and that, in reality, no variable may consistently predict prognosis across the broader FS population. It is also possible that only a small subset of individuals presents values markedly different from the overall sample, meaning that the prognostic factors identified in the main analysis may primarily reflect these atypical cases. This highlights the need for a thorough assessment of the individuals’ nonbiological profiles, as high levels of psychological distress or specific traits could significantly influence the prognosis.

Conversely, when SPADI disability was considered as the outcome, state anxiety remained a significant prognostic factor even after the outlier analysis. This result suggests that the patient’s subjective experience of their condition significantly impacts disability, underlining the importance of education and reassurance as key therapeutic strategies to improve disability outcomes. Moreover, this finding highlights the need for a comprehensive assessment of the patient’s current psychological state, as it may critically influence treatment efficacy.

However, both these findings reinforce the necessity of adopting a person‐centered treatment approach, tailored to the unique characteristics of each individual that may condition their response to intervention.

### 4.3. Implication for Clinical Practice

Clinicians should reconsider the current assessment protocols for individuals with FS, which traditionally focus on range of motion and pain severity while often underestimating the psychological dimension of the condition [26, 63]. In line with contemporary evidence‐based practice, a broader assessment of nonbiological factors, particularly anxiety‐related constructs, should be incorporated into routine clinical evaluation. The present findings suggest that both trait and state anxiety are relevant to pain and disability trajectories, highlighting the importance of psychological screening early in the clinical course. Although other factors, such as fear‐avoidance beliefs and health‐related quality of life, were not prognostic in this cohort, previous literature indicates their potential relevance in musculoskeletal conditions more broadly [10, 12, 13], supporting their consideration within a comprehensive assessment framework.

Furthermore, emerging evidence from biochemical, neuroimmunological, and endocrine research suggests that FS may be influenced by broader systemic processes, including metabolic dysfunction [64, 65]. From this perspective, FS can be interpreted as an epiphenomenon of a systemic disease with profound implications for metabolic and psychological domains, as well as physical function. Such an approach aligns more closely with patients’ lived experiences [15] and clinical expectations [66] and may facilitate more individualized, person‐centered management strategies.

### 4.4. Implication for Research

Researchers should design structured studies to assess the utility and efficacy of adjunctive interventions combined with physiotherapy, aiming to optimize the management of nonbiological variables in FS individuals. Although the present study did not find significant prognostic value for variables such as catastrophizing, central sensitization, fear‐avoidance, and insomnia, these correlations warrant further investigation. Future research should consider a broader range of outcomes, such as an objective range of motion and health‐related quality of life, while utilizing more ample and diverse clinical samples. Ideally, these should include cohorts with more compromised baseline psychological profiles, central sensitization, and sleep disturbances to avoid potential floor effects. Such investigations are essential to determine whether different phenotypes require tailored rehabilitative pathways. Lastly, different dependent variables (such as self‐efficacy, pain beliefs, and perception and depression) should be considered in future investigations. From a modern, person‐centered perspective, this approach would allow clinicians to stratify care, ensuring that rehabilitation is not only focused on physical recovery but is also specifically calibrated to the patient’s unique psychological and systemic needs.

To advance this stratified perspective, future prognostic designs should also improve phenotyping of both situational and dispositional factors. Future prognostic studies should routinely include STAI‐state and trait anxiety alongside validated personality profiling to capture both situational distress and dispositional vulnerability, and to test whether personality moderates the relationship between anxiety and clinical trajectories in FS. Where feasible, pairing this psychosocial phenotyping with immune‐metabolic measures (e.g., markers of low‐grade inflammation and metabolic dysregulation) would allow direct testing of the hypothesized neuroimmune–immunometabolic pathways and could strengthen stratified, person‐centered rehabilitation approaches. A further key direction for future studies lies in moving beyond individual predictors toward the construction of integrated prognostic models capable of accounting for multiple interacting variables.

### 4.5. Limitations

While the present investigation is supported by a robust methodology, clear and comprehensive reporting, and a rigorous statistical perspective, certain limitations must be acknowledged.

First, although our recruited sample accurately represents the typical clinical demographic of FS (predominantly 55‐year‐old women with a high prevalence of metabolic comorbidities like diabetes and thyroid disorders), some specific characteristics may limit broader generalization. Specifically, the majority of participants was affected in their dominant arm and did not lead strictly sedentary lifestyles. This profile may affect the external validity of our findings when applied to the entire FS population, which often exhibits lower activity levels and contralateral limb involvement [1, 3].

Regarding the statistical analysis, the decision not to include confounding variables such as age, gender, and specific comorbidities in the regression models may have influenced the precision of our prognostic estimates. However, this choice was necessitated by the limited sample size available for this study, which precluded the inclusion of additional covariates without risking model overfitting. Methodologically, the absence of a home exercise diary prevented us from objectively verifying compliance with the prescribed self‐management program; moreover, variations in the intensity of pharmacological management and rehabilitation adjustments based on tissue irritability may have introduced additional variability.

Additionally, several potentially significant prognostic variables were not captured in this study, including baseline depression, pain beliefs, and workers’ compensation status. The exclusion of these factors limits our ability to fully elucidate the mechanisms underlying perceived pain and disability in FS. Finally, the study did not evaluate the impact of range of motion, health literacy, or disease staging as independent variables, nor did it assess range of motion and health‐related quality of life as primary dependent outcomes.

## 5. Conclusion

Our findings demonstrate that trait anxiety and state anxiety hold a statistically significant prognostic value for short‐term pain and disability outcomes, respectively, in individuals with FS. Conversely, other variables—including pain catastrophizing, fear‐avoidance behavior, central sensitization, and insomnia—were not significantly associated with clinical outcomes in this cohort. These results provide clinically meaningful insights for clinicians managing FS, providing a more nuanced understanding of prognosis within a conservative care context. Such evidence is crucial for establishing realistic expectations, strengthening the therapeutic alliance, fostering effective interdisciplinary collaboration, and tailoring conservative interventions, strictly adhering to the principles of person‐centered care.

## Author Contributions

Concept/idea: Fabrizio Brindisino.

Research design: Fabrizio Brindisino.

Writing: Fabrizio Brindisino, Santiago Navarro Ledesma, Germanna Medeiros Barbosa, Filip Struyf.

Data collection: Fabrizio Brindisino, Fabrizio Pulina, Elena Silvestri, Alessio Fioretti.

Data analysis: Fabrizio Brindisino.

Project management: Fabrizio Brindisino.

Consultation (including review of manuscript before submitting): Fabrizio Brindisino, Elena Silvestri, Alessio Fioretti, Fabrizio Pulina, Jacopo Conteduca, Santiago Navarro Ledesma, Germanna Medeiros Barbosa, Filip Struyf.

## Funding

No funding was received for this manuscript.

## Disclosure

All the authors approved the final manuscript.

## Ethics Statement

Ethical approval was obtained from the Ethics Committee of the University of Molise (Italy) with the registration number Prot. *n*. 06/2025 from 19/06/2025. All the study‐related procedures were performed according to the principles of the Declaration of Helsinki. Informed consent to participate was provided by all included participants.

## Conflicts of Interest

The authors declare no conflicts of interest.

## Supporting Information

Additional supporting information can be found online in the Supporting Information section.

## Supporting information


**Supporting Information 1** Supporting Information 1. INFORMATION LETTER.

**Supporting Information 2** Supporting Information 2. VARIANCE INFLATION FACTORS for pain and disability.

**Supporting Information 3** Supporting Information 3. OUTLIER ANALYSIS for pain and disability.

## Data Availability

The data that support the findings of this study are available in the supporting information of this article.
