# Supplementary material for: Psychological Factors, Central Sensitization, and Insomnia as Potential Prognostic Factors for Short‐Term Pain and Disability in Patients With Frozen Shoulder: A Multicentric Prospective Single‐Cohort Study
Source: Pain Res Manag. 2026 Apr 13;2026:6298409. doi: 10.1155/prm/6298409 (PMC13071860; doi:10.1155/prm/6298409)
Supplement: Supplementary file 1 — Supporting Information 1 Supporting Information 1. INFORMATION LETTER. [file PRM-2026-6298409-s001.docx]

**SUPPLEMENTARY FILE 1 – INFORMATION LETTER**

**Psychological factors, central sensitization and insomnia as potential prognostic factors for short-term pain and disability in patients with frozen shoulder. A multicentric prospective single-cohort study**

Dear Participant,

We are researchers from the XXXXXX, and we are currently conducting a research project dedicated to individuals affected by Frozen Shoulder.

**Objective**: The aim of this study is to better understand whether specific psychological factors can predict prognostic outcomes at a three-month follow-up.

**Eligibility Criteria**: To participate, you must:

- a) Have received a diagnosis of Frozen Shoulder from a healthcare professional;
- b) Be proficient in the Italian language.

**Voluntary Participation and Anonymity**: Participation in this study is entirely VOLUNTARY and ANONYMOUS. No personally identifiable information will be collected. At the beginning of the survey, you will be asked for basic demographic data to characterize the study sample; however, it will be impossible to link your identity to your responses. All data will be anonymized and processed independently by a statistician.

**Data Usage**: Data will be used in an aggregated format for scientific publications or conference presentations aimed at improving the clinical management of individuals with Frozen Shoulder.

**PLEASE NOTE**:

- a) Complete the questionnaires only after providing your informed consent by selecting "YES" below;
- b) You will be contacted via email by the same researcher in 3 months for a follow-up assessment;
- c) You may stop the survey at any time without consequences and withdraw from the study without providing any justification;
- d) After answering all questions, ensure you click the "Submit" button to send your responses.

Thank you for your time and contribution.

XXXXXXX

For any information, please contact: [XXXXXXX](mailto:fabrizio.brindisino@unimol.it)

Informed Consent by answering the following question, you are providing your implicit informed consent to participate in this study.

**Do you wish to participate in the survey?**

- ▹ YES, I wish to proceed to the questionnaire.
- ▹ NO, thank you.
