# Supplementary material for: Psychological Factors, Central Sensitization, and Insomnia as Potential Prognostic Factors for Short‐Term Pain and Disability in Patients With Frozen Shoulder: A Multicentric Prospective Single‐Cohort Study
Source: Pain Res Manag. 2026 Apr 13;2026:6298409. doi: 10.1155/prm/6298409 (PMC13071860; doi:10.1155/prm/6298409)

**SUPPLEMENTRY FILE 2 – VARIANCE INFLATION FACTORS**

**SPADI pain:**

| **VARIABLE** | **VIF** |
| --- | --- |
| PCS | 1.61 |
| STAI trait | 3.10 |
| STAI state | 3.13 |
| FABQ physical activity | 1.38 |
| FABQ work | 1.48 |
| CSI | 1.77 |
| ISI | 1.80 |

**Acronyms:** SPADI, Shoulder Pain and Disability Index**;** VIF, variance inflation factors; PCS, Pain Catastrophizing Scale; STAI, State–Trait Anxiety Inventory; FABQ, Fear Avoidance Beliefs Questionnaire; CSI, Central Sensitization Inventory; ISI, Insomnia Severity Index

**Verification of underlying assumptions:**


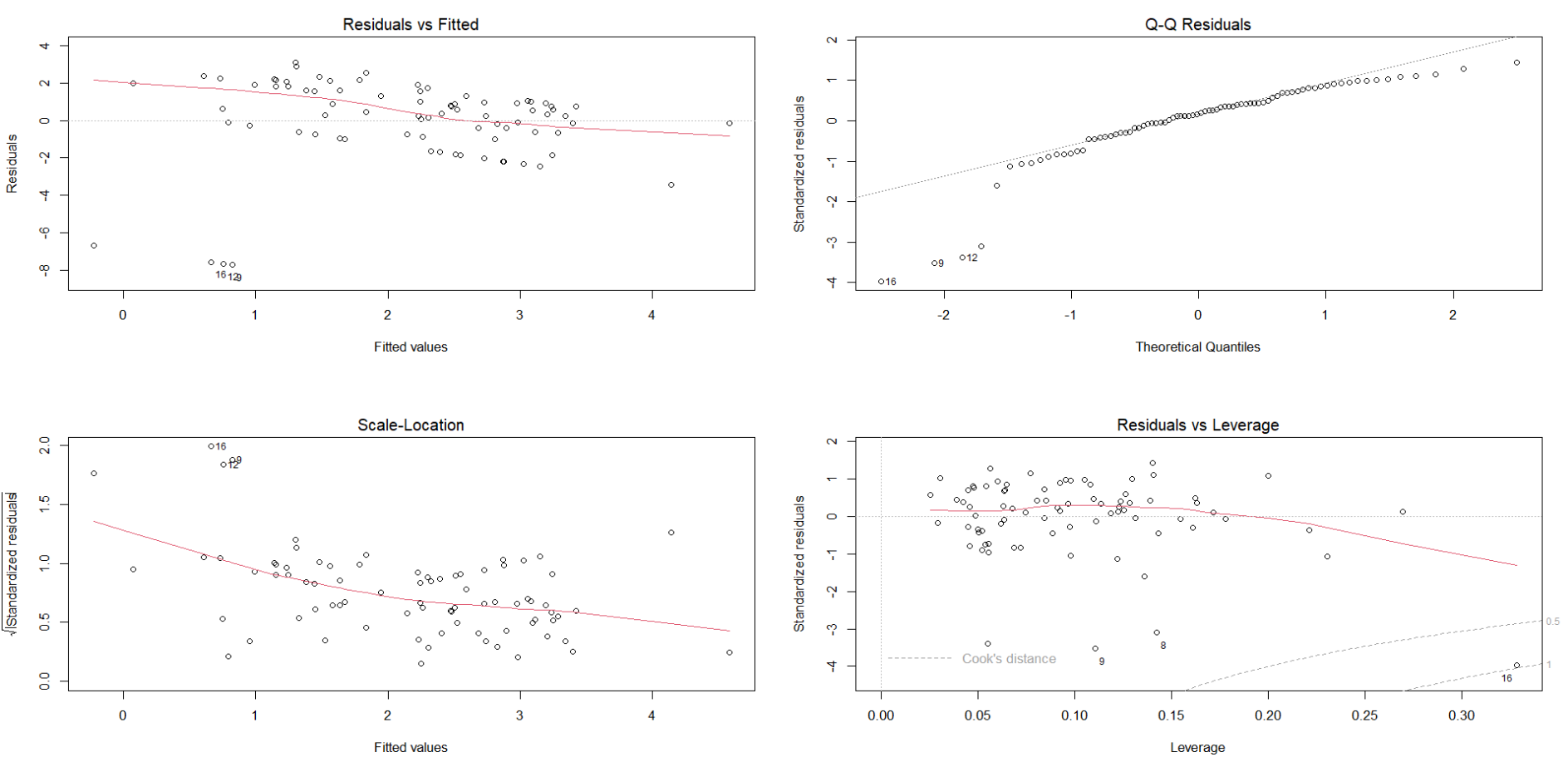


**Cook’s distance for the identification of the influential observations**
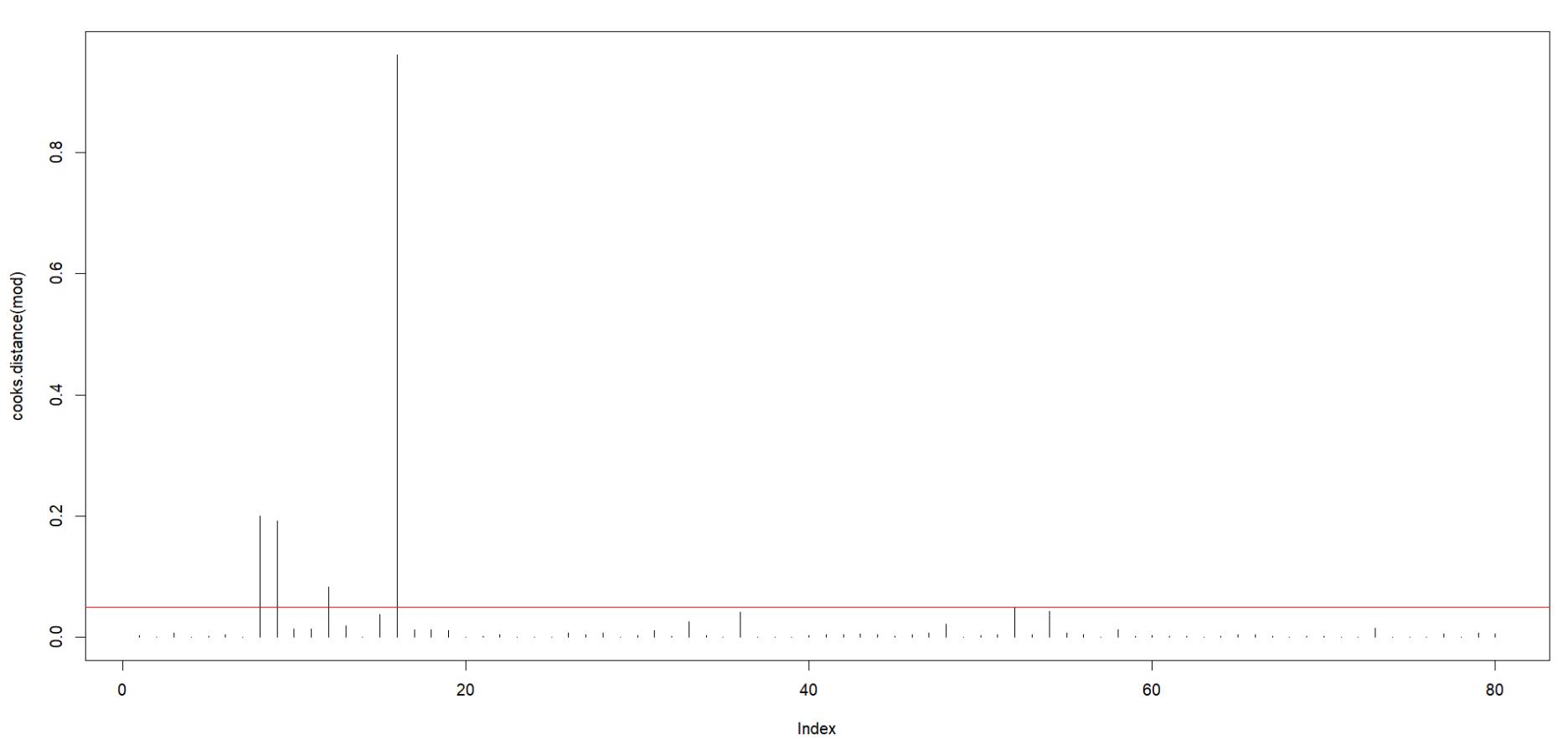


**SPADI disability:**

| **VARIABLE** | **VIF** |
| --- | --- |
| PCS | 1.60 |
| STAI trait | 3.14 |
| STAI state | 3.18 |
| FABQ physical activity | 1.38 |
| FABQ work | 1.58 |
| CSI | 1.75 |
| ISI | 1.58 |

**Acronyms:** SPADI, Shoulder Pain and Disability Index; VIF, variance inflation factors; PCS, Pain Catastrophizing Scale; STAI, State–Trait Anxiety Inventory; FABQ, Fear Avoidance Beliefs Questionnaire; CSI, Central Sensitization Inventory; ISI, Insomnia Severity Index

**Verification of underlying assumptions:**


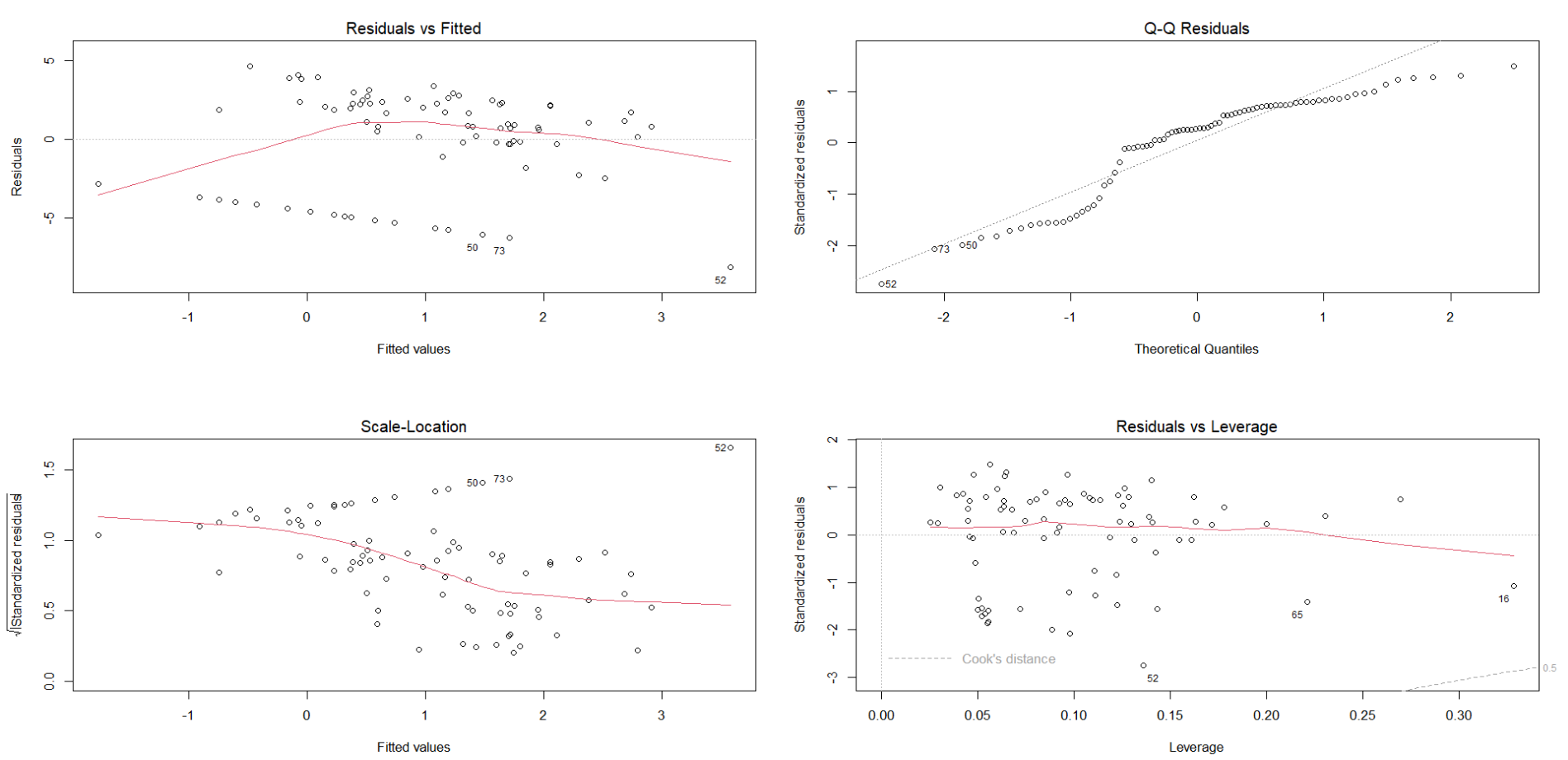


**Cook’s distance for the identification of the influential observations**


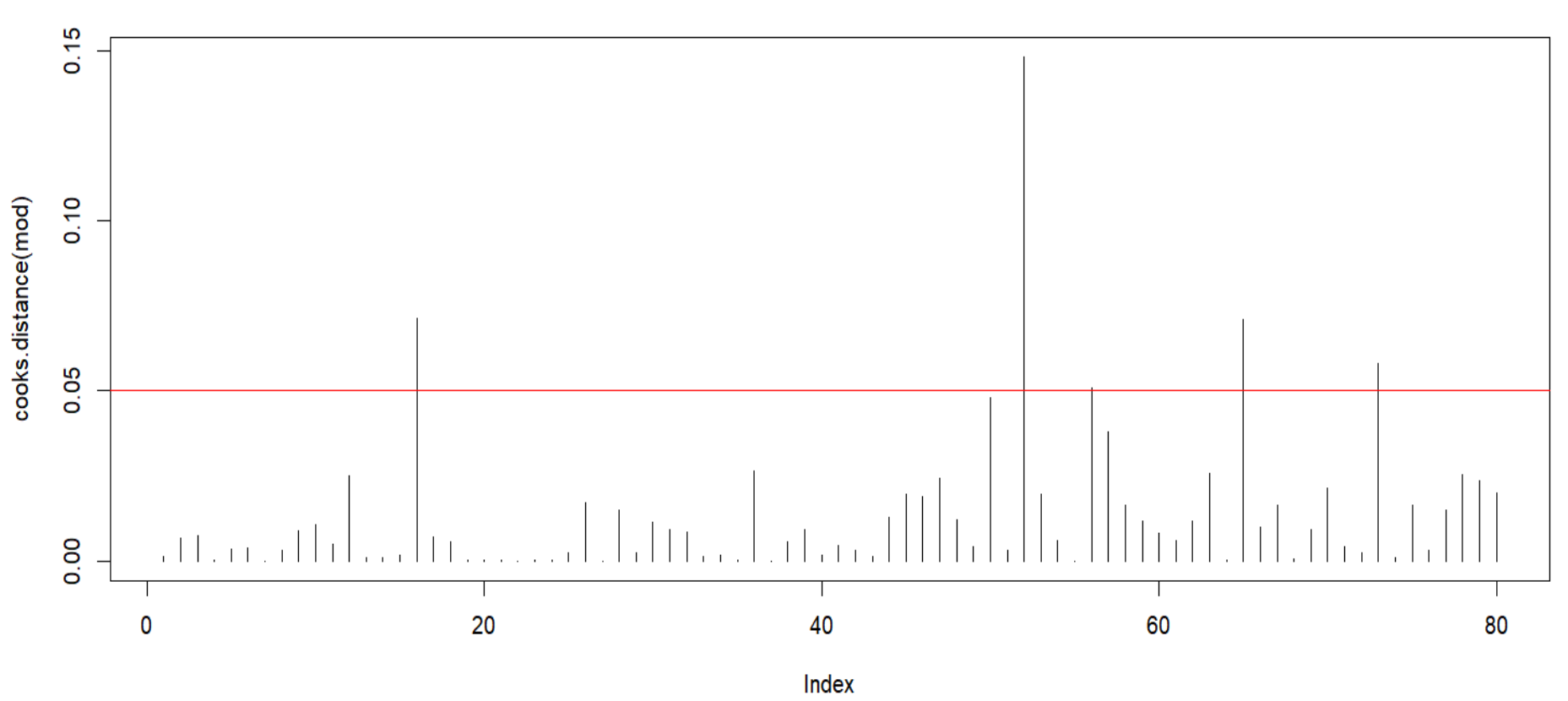

Supplement: Supplementary file 2 — Supporting Information 2 Supporting Information 2. VARIANCE INFLATION FACTORS for pain and disability. [file PRM-2026-6298409-s002.docx]
