# Supplementary material for: Psychological Factors, Central Sensitization, and Insomnia as Potential Prognostic Factors for Short‐Term Pain and Disability in Patients With Frozen Shoulder: A Multicentric Prospective Single‐Cohort Study
Source: Pain Res Manag. 2026 Apr 13;2026:6298409. doi: 10.1155/prm/6298409 (PMC13071860; doi:10.1155/prm/6298409)
Supplement: Supplementary file 3 — Supporting Information 3 Supporting Information 3. OUTLIER ANALYSIS for pain and disability. [file PRM-2026-6298409-s003.docx]

**SUPPLEMENTARY FILE 3 – OUTLIER ANALYSIS**

**Sensitivity analysis of SPADI pain deleting outliers**

| **VARIABLE** | **MULTIVARIABLE ANALYSIS** | |
| --- | --- | --- |
|  | **exp(coefficients)** | **P – value** |
| PCS | 1.00 (0.98 to 1.01) | 0.83 |
| STAI trait | 1.03 (0.95 to 1.11) | 0.52 |
| STAI state | 0.97 (0.91 to 1.03) | 0.29 |
| FABQ physical activity | 1.02 (0.97 to 1.08) | 0.33 |
| FABQ work | 0.99 (0.96 to 1.02) | 0.41 |
| CSI | 1.00 (0.97 to 1.02) | 0.70 |
| ISI | 1.02 (0.96 to 1.08) | 0.52 |

**Acronyms*.*** *PCS, Pain Catastrophizing Scale; STAI, State–Trait Anxiety Inventory; FABQ, Fear Avoidance Beliefs Questionnaire; CSI, Central Sensitization Inventory; ISI, Insomnia Severity Index*

**Sensitivity analysis of SPADI disability deleting outliers**

| **VARIABLE** | **MULTIVARIABLE ANALYSIS** | |
| --- | --- | --- |
|  | **exp(coefficients)** | **P – value** |
| PCS | 0.99 (0.95 to 1.02) | 0.41 |
| STAI trait | 1.10 (0.91 to 1.33) | 0.29 |
| STAI state | **0.86 (0.74 to 0.99)** | **0.04** |
| FABQ physical activity | 1.07 (0.95 to 1.20) | 0.28 |
| FABQ work | 0.97 (0.90 to 1.03) | 0.32 |
| CSI | 1.02 (0.96 to 1.09) | 0.53 |
| ISI | 1.04 (0.90 to 1.19) | 0.61 |

**Acronyms*.*** *PCS, Pain Catastrophizing Scale; STAI, State–Trait Anxiety Inventory; FABQ, Fear Avoidance Beliefs Questionnaire; CSI, Central Sensitization Inventory; ISI, Insomnia Severity Index*
